# In-depth Characterization of Firefly Luciferase as a Reporter of Circadian Gene Expression in Mammalian Cells

**DOI:** 10.1177/0748730416668898

**Published:** 2016-10-10

**Authors:** Kevin A. Feeney, Marrit Putker, Marco Brancaccio, John S. O’Neill

**Affiliations:** MRC Laboratory of Molecular Biology, Francis Crick Avenue, Cambridge, UK

**Keywords:** firefly luciferase, bioluminescent reporter, clock gene, mammalian circadian rhythms, enzyme kinetics

## Abstract

Firefly luciferase (Fluc) is frequently used to report circadian gene expression rhythms in mammalian cells and tissues. During longitudinal assays it is generally assumed that enzymatic substrates are in saturating excess, such that total bioluminescence is directly proportional to Fluc protein level. To test this assumption, we compared the enzyme kinetics of purified luciferase with its activity in mammalian cells. We found that Fluc activity in solution has a lower Michaelis constant (K_m_) for luciferin, lower temperature dependence, and lower catalytic half-life than Fluc in cells. In consequence, extracellular luciferin concentration significantly affects the apparent circadian amplitude and phase of the widely used PER2::LUC reporter in cultured fibroblasts, but not in SCN, and we suggest that this arises from differences in plasma membrane luciferin transporter activity. We found that at very high concentrations (>1 mM), luciferin lengthens circadian period, in both fibroblasts and organotypic SCN slices. We conclude that the amplitude and phase of circadian gene expression inferred from bioluminescence recordings should be treated with some caution, and we suggest that optimal luciferin concentration should be determined empirically for each luciferase reporter and cell type.

Firefly luciferase (Fluc) catalyzes the Mg·ATP-dependent oxidation of luciferin (LH_2_) through an enzyme-bound intermediate (Fluc·LH_2_-AMP) to release light in a 2-step reaction (Deluca, 1976):

$$\mathrm{Fluc}+{\mathrm{LH}}_{2}+\mathrm{ATP}-{\mathrm{Mg}}^{2+}<=>\mathrm{Fluc}\cdot {\mathrm{LH}}_{2}-\mathrm{AMP}+{\mathrm{PP}}_{i}-{\mathrm{Mg}}^{2+}$$

$$\mathrm{Fluc}\cdot {\mathrm{LH}}_{2}-\mathrm{AMP}+O_{2}=>\mathrm{Fluc}+\mathrm{oxyluciferin}+\mathrm{AMP}+{\mathrm{CO}}_{2}+\mathrm{photon}.$$

Fluc is the best-characterized bioluminescent protein and is used widely as a tool for reporting gene expression levels within living cells (Gelmini et al., 2000). In circadian research, Fluc is frequently expressed under the control of circadian regulated promoter sequences to report oscillations in transcriptional activity, for example, Per2:Luc (Ueda et al., 2002). As a fusion protein, Fluc also has been used most successfully to report the activity of circadian-regulated proteins, for example, PER2::LUC (Yoo et al., 2004).

Pioneering investigations into the phenomenon of bioluminescence conducted by McElroy, White, Seliger, and coworkers led to the discovery of the protein responsible for light emission by fireflies as well as its substrates (White et al., 1969; DeLuca and McElroy, 1974; Deluca, 1976), setting the stage for the detailed characterization of Fluc mechanism elucidated during subsequent decades (Nakatsu et al., 2006; Inouye, 2010). Critical to its utility as a reporter of circadian gene expression are the enzyme kinetics of Fluc activity, its turnover, and catalytic inactivation. If Fluc folded more slowly, for instance, or had higher catalytic stability, this would significantly reduce the temporal resolution with which changes in gene expression could be detected in longitudinal assays (Millar et al., 1992; Gelmini et al., 2000; McClung, 2006).

In terms of enzymatic activity, the turnover number of luciferase is reportedly rather low (Ignowski and Schaffer, 2004; Marques and Esteves da Silva, 2009), with quite poor thermal stability in solution (Kitayama et al., 2003). Expressed in cells, however, luciferase protein is quite stable (half-life of many hours) and accumulates to high levels in the absence of destabilization tags (Leclerc et al., 2000; Gupta et al., 2011). Seminal publications by Kay and Millar (Millar et al., 1992; Millar et al., 1995) identified the utility of Fluc as a genetically encoded reporter for circadian rhythms and revealed that, critically, the catalytic stability of Fluc is significantly lower than its protein stability, being sufficiently short (<6 h) to enable real-time reporting of circadian gene expression regulation (McClung, 2006; Jacobshagen et al., 2008). This “catalytic exhaustion” of Fluc occurs stochastically and likely involves the formation of a luminescently inactive, irreversibly oxidized, luciferase-oxyluciferin complex (Gandelman et al., 1994; Branchini et al., 1997).

It is generally assumed that substrate concentration, in the context of its modern application in circadian research, remains in saturating excess, meaning that light emission varies linearly with the concentration of active Fluc. Although Fluc is an intensely studied enzyme, a range of kinetic constants have been reported (Branchini et al., 1999; Ignowski and Schaffer, 2004; Inouye, 2010), and in very few cases have those biochemical measurements been compared directly with the activity of luciferase in cells (Ignowski and Schaffer, 2004; van Ooijen et al., 2011). To better understand how much can meaningfully be inferred from bioluminescence assays that are performed routinely in circadian research, we asked what biologically relevant factors contribute to Fluc activity in solution, compared with its activity when expressed heterologously in mammalian cells and SCN organotypic slices.

## Materials and Methods

All reagents were purchased form Sigma-Aldrich (St. Louis, MO) unless otherwise stated. Potassium luciferin salt was purchased from Biosynth (Staad, Switzerland) (≥99.5% purity), and the same batch was used throughout.

### Luciferase Assays in Solution

QuantiLum is purified recombinant firefly luciferase (Promega, Madison, WI; E1701). QuantiLum was used at a concentration of 10 nM throughout, although we confirmed that substrate is in saturating excess under default buffer conditions, since at no point in a 4-log dilution series of luciferase concentration (1 nM to 1 µM) did light emission depart from linearity (not shown). The default buffer contained final concentrations of 30 mM HEPES pH 7.4, 10 mM β-mercapto-ethanol, 1 mM ATP pH 7.4, 1 mM potassium luciferin, 10 mM MgSO_4_, and 1 mg/mL bovine serum albumin, but these were varied in composition as reported in the text. Bioluminescence was measured using a Tecan Spark 10M plate reader. For each analysis, 90 µL reaction buffer per well was set up in 96-well plates and allowed to equilibrate to incubation temperature for 15 min. Reactions were then initiated by automated injection of 10 µL of either 10 mM ATP or luciferin into the reaction buffer. Automated injections were performed at 200 µL/sec, and measurement of light emission began 100 msec afterward. Pyrophosphatase (NEB, Ipswich, MA) was used at a 1000-fold dilution from a 100 U/mL stock. CoASH was dissolved in 30 mM HEPES in a 10 mM stock solution.

### Mammalian Cell Culture

Human U2OS cells were obtained from ATCC (Teddington, UK) and stably transfected with pGL4.20 constructs (Promega) to express Fluc either constitutively (SV40:Luc) or under the control of the Per2 or Bmal1 promoter, as described previously (Burke et al., 2015). PER2::LUC fibroblasts were isolated from adult mouse lung tissue and immortalized as described previously (Causton et al., 2015). U2OS and fibroblast cells were cultured in Dulbecco’s modified eagle medium with Glutamax (Thermo Fisher, Waltham, MA), supplemented with 100 U penicillin/mL and 100 µg/mL streptomycin, as well as 10% FetalClone II or FetalClone III serum (HyClone, Thermo Fisher), respectively. Prior to circadian bioluminescence recording, cells were maintained under 12-h:12-h 32:37 °C temperature cycles for at least 1 week to ensure maximum intercellular synchrony at the beginning of each experiment.

### Organotypic SCN Slice Culture and Recording

Organotypic SCN slices from p12-p14 mouse pups were isolated and transduced with 1 µL of AAV expressing GCaMP3 under the control of human Synapsin1 promoter (purchased from Penn Vector Core, Philadelphia, PA), as previously described (Brancaccio et al., 2013). PER2::LUC amplitude was analyzed using a 48-h window, immediately prior to and after the addition of extra luciferin. Organotypic SCN bioluminescence and fluorescence were performed using an LV200 microscope system (Olympus, Tokyo, Japan).

### Cellular Bioluminescence Assays

Bioluminescence recording medium was identical to DMEM except that 20 mM HEPES pH 7.4 and 2% B27 (Thermo Fisher) were included, along with a range of luciferin concentrations (as described), and the concentration of sodium bicarbonate concentration was reduced to 0.035%. Assay of cells expressing Fluc constitutively was performed in a Tecan (Zurich, Switzerland) Spark 10M plate reader. Recordings of PER2::LUC fibroblasts and recordings that included temperature cycles were performed in an ALLIGATOR (Cairn Research, Faversham, UK). We did not explicitly control for the effect of changes in luciferin’s counter-ion (K^+^) concentration during our assays because, similar to Noguchi et al. (2013), we observed no effect on cellular bioluminescence of 1 to 10 mM additional extracellular [K^+^] (not shown). For determination of K_m_ with respect to oxygen, gas was allowed to equilibrate for 4 h after each transition in gas mixture, with extracellular 1 mM luciferin present throughout, before bioluminescence was measured.

### Analysis

Circadian bioluminescence data were detrended to remove baseline changes and then fitted with a damped sine wave in order to determine circadian period, amplitude, and phase, as described by Hirota et al. (2008). This and all other statistical analyses were performed using Graphpad Prism (San Diego, CA). To determine Fluc enzymatic half-life in solution, under conditions of saturating substrate excess, a 1-phase exponential decay curve was fit to the first 60 min of luminescence recorded from each well.

## Results

### Enzyme Kinetics of Fluc in Solution

**Figure 1.**
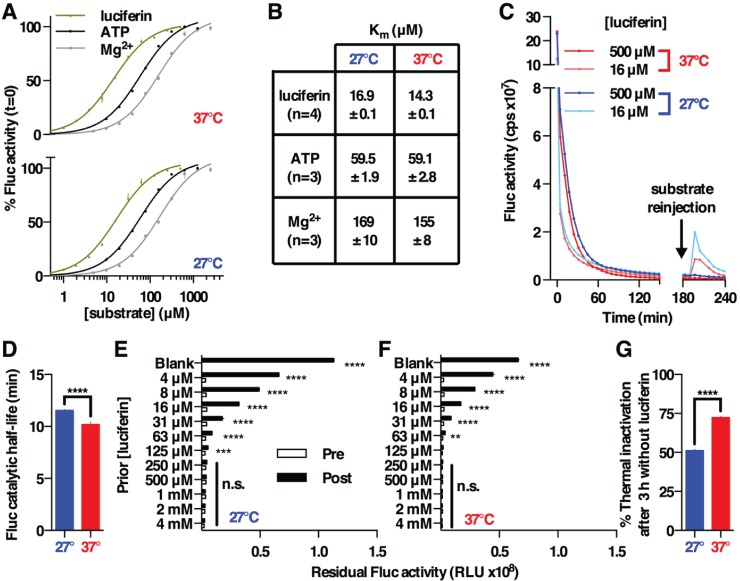
Enzyme kinetics of recombinant Fluc in solution. (A) Data points show normalized Fluc activity (mean ± SEM) with Michaelis-Menten fit (solid line, *R*^2^ > 0.99). (B) Fluc K_m_ values (mean ± SEM) derived from (A); replicate numbers are reported for each substrate. (C) Representative experimental data from a substrate dilution series (luciferin in this case), where Fluc activities at t = 0 were used to derive A and B (mean ± SEM, *n* = 4). The decay in bioluminescence at 500 µM (saturating) luciferin was fit with a 1-phase exponential decay function (*R*^2^ > 0.99) to give the Fluc catalytic half-life values reported in (D), *p* < 0.0001, unpaired *t* test. After 3 h, ATP and luciferin were reinjected to 1 mM final concentration (black arrow, C), with the Fluc activity before and after reinjection being reported in (E) and (F), which both showed a significant interaction between injection and prior luciferin concentration by 2-way ANOVA (*p* < 0.0001). Significance by Sidak’s multiple comparisons test is also reported (**p < 0.01, ***p < 0.001, ****p < 0.0001, n.s. *p* > 0.5). (G) Calculated % Fluc thermal inactivation after 3 h in the absence of luciferin, *p* < 0.0001, unpaired *t* test.

We first determined the activity of Fluc for 2 of its initial substrates, ATP and luciferin, by measuring how the initial reaction rate varied as a function of substrate concentration. This is reported as the Michaelis constant (K_m_), the substrate concentration at which half the maximal enzyme activity (V_max_) is observed (Michaelis et al., 2011). Although Mg^2+^ is not strictly a substrate, we also measured the K_m_ for Mg^2+^ since it is an essential cofactor for ATP and its intracellular availability is dynamically regulated (Feeney et al., 2016). We performed these assays at 27 °C and 37 °C to reflect the range of temperatures over which Fluc has been used in mammalian cells. The resultant curves conformed to standard Michaelis-Menten (MM) kinetics (Fig. 1A), producing values for K_m_ within the expected range (Fig. 1B). We were surprised to observe, however, that K_m_ was compensated against a 10 °C change in temperature with respect to all 3 substrates (see Figure 1C for representative example of experimental data).

We determined the K_m_ of Fluc for ATP and Mg^2+^ to be more than 10-fold lower than the lowest estimates of their intracellular concentration in unstressed cells (1-5 mM and 15-30 mM, respectively) (Ebel and Gunther, 1980; Gribble et al., 2000; Dennis et al., 2001; de Baaij et al., 2015). We therefore deem it unlikely that changes in ATP and Mg^2+^ within living cells might conceivably lead to appreciable changes in bioluminescence, and it is thus reasonable to assume that Mg·ATP is in saturating excess during cellular circadian bioluminescence assays.

We next considered the inactivation kinetics of Fluc. Under conditions of saturating substrate excess (e.g., 500 µM luciferin, Figure 1C), the decay in bioluminescence over time exhibited 1-phase exponential decay (*R*^2^ > 0.99). The significant but modest effect of reaction temperature on Fluc enzymatic stability (Fig. 1D), coupled with increased stability of bioluminescence at lower substrate concentrations (compare 16 µM with 500 µM luciferin in Figure 1C, for example), prompted us to consider whether any relationship existed between enzymatic turnover and the rate at which Fluc becomes catalytically exhausted (irreversibly inactive).

To test this, after 3 h of preincubation with varying luciferin concentrations, we supplemented each reaction well with additional ATP and luciferin by reinjection (to 1 mM final concentration). This allowed us to measure how much Fluc activity persisted as a function of prior catalytic turnover at 2 different temperatures (Fig. 1C, E, F). At 27 °C we observed that no significant residual Fluc activity was detected after prior incubation with luciferin concentrations ≥250 µM (Fig. 1E), allowing us to interpolate that the average Fluc molecule catalyzes approximately 20,000 reactions before its inactivation under these conditions. By comparing the integrated light emission before and after substrate reinjection, with Fluc activity in the presence or absence of luciferin before reinjection (500 µM vs. blank), we determined that roughly half (51.2% ± 0.4%) of the decay in Fluc activity was directly attributable to thermal inactivation over 3 h, with the remainder being due to “catalytic exhaustion” (Gandelman et al., 1994). At 37 °C, no significant residual Fluc activity was detected where prior luciferin concentrations had been 125 µM or higher (Fig. 1F), meaning that the average Fluc molecule performed ~10,000 reactions before inactivation under these conditions. This reduction in total number of reactions, between 37 and 27 °C, was largely attributable to a temperature-dependent decrease in thermal stability, since only 27.6% ± 0.4% of initial Fluc activity remained after a 3-h incubation in the absence of luciferin (Fig. 1G). These observations imply that the thermal stability of luciferase is not temperature compensated (Q10 for decay constant = 1.86 ± 0.03), in concordance with prior reports (Kitayama et al., 2003).

Based on the initial reaction rates (at t = 0), we calculated a turnover number (k_cat_) of 38 sec^-1^ with respect to luciferin. This number is not particularly relevant to the steady-state conditions under which Fluc is active within cells, however, since it derives from the assembly of a quaternary complex from a tertiary one (comprising Fluc already complexed with Mg^2+^ and ATP). We thought it informative therefore to also calculate the apparent k_cat_ (^app^k_cat_) based on Fluc activity at t = 300 sec, to more closely model the steady-state conditions facing newly synthesized Fluc within cells; that is, where some product has accumulated, substrate remains in excess, and turnover is a function of both Mg·ATP and luciferin binding as well as the decay of activated luciferin and subsequent product release (Cleland, 2009). Using the Michaelis-Menten model, we determined that ^app^k_cat_ = 9.3 ± 0.1 sec^-1^. By integrating the total light emission for reactions that proceeded to catalytic exhaustion, compared with the efficiency of detection and numbers of molecules involved, we calculated an approximate turnover of 9.5 reactions per Fluc per second under the same conditions. Since these values agree closely with each other, when all substrates remain in excess it is likely that an active Fluc molecule catalyzes the emission of roughly 10 photons per second under the quasi-steady-state conditions within cells.

In cells, the activity of many proteins is affected by changes in metabolic activity. As shown above, the K_m_ of Fluc for Mg·ATP is sufficiently low and its turnover is so slow that biologically plausible changes in [Mg·ATP] are unlikely to affect Fluc activity during cellular bioluminescence assays. Fluc activity is also affected by coenzyme A (CoASH) and its product, inorganic pyrophosphate (PPi) (Marques and Esteves da Silva, 2009). CoASH and PPi are reported, variously, as being allosteric regulators of Fluc or substrates that react with an inhibitory side-product of the main reaction (Gandelman et al., 1994; Fraga et al., 2003; Fontes et al., 2008; da Silva and da Silva, 2011). We found, however, that although Fluc activity was significantly affected by physiologically relevant changes in CoASH and PPi, the actual effect size was rather modest under our assay conditions. (Suppl. Fig. S1). Thus, in the absence of large-scale variations in these parameters, they are unlikely to have major effects on Fluc activity in cells under excess substrate conditions.

### Activity of Fluc in Mammalian Cells

**Figure 2. fig2-0748730416668898:**
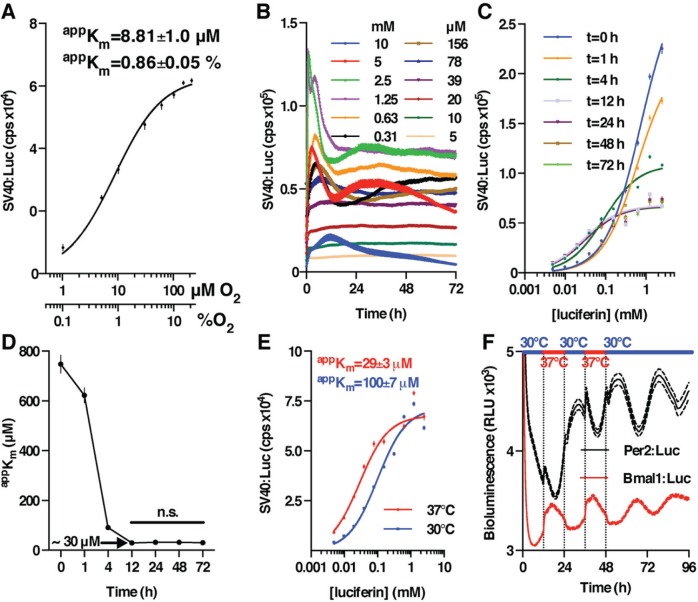
Characterization of Fluc activity expressed constitutively in human U2OS cells. (A) Apparent K_m_ of Fluc for oxygen (mean ± SEM, *n* = 96) with MM fit (solid line, *R*^2^ = 0.81). (B) Fluc activity over 3 days in cells incubated with a range of luciferin concentrations (mean ± SEM, *n* = 7). (C) MM fit (solid line, *R*^2^ > 0.9) to determine ^app^K_m_ for luciferin at different incubation times. (D) After 12 h of incubation with luciferin, ^app^K_m_ for luciferin does not change significantly (n.s., *p* > 0.99). (E) After 12 h of incubation with luciferin, calculated ^app^K_m_ is significantly greater at 30 °C than at 37 °C (sum-of-squares *F* test, *p* < 0.0001), mean ± SEM (*n* = 7); solid line shows MM fit (*R*^2^ > 0.9). (F) Fluc activity increases at 30 to 37 °C transition when expressed under antiphasic circadian promoters, mean ± SEM (*n* = 8), performed using extracellular 300 µM luciferin. Also see Suppl. Fig. S2.

For technical reasons, we could not measure the K_m_ of Fluc with respect to oxygen, its other substrate, in solution. We were successful, however, in measuring the apparent K_m_ (^app^K_m_) under steady-state conditions in U2OS cells expressing Fluc constitutively from the SV40 promoter. At 37 °C, we found that ^app^K_m_ was less than 1% oxygen partial pressure (pO_2_) (Fig. 2A). To saturate an enzyme’s active site, substrate concentrations should be more than 10-fold greater than their respective K_m_ so that their availability is not rate limiting (Cleland, 2009; Michaelis et al., 2011). The ^app^K_m_ of Fluc for oxygen is significant, therefore, since it confirms an unstated assumption: that oxygen availability is not rate limiting under most experimental conditions where circadian bioluminescence recordings are performed (atmospheric pO_2_ = 21%). In tissues in vivo, however, the pO_2_ can fall below 2% (Leach and Treacher, 2002; Marcinek et al., 2003), and this should be borne in mind when one is interpreting data gleaned from transgenic luciferase-expressing rodent models.

We next investigated how the ^app^K_m_ of Fluc, with respect to luciferin, compared with the values we measured in solution. U2OS cells constitutively expressing Fluc were incubated with a range of extracellular luciferin concentrations, and the resultant bioluminescence was measured (Fig. 2B, see also Suppl. Fig S2A). Bioluminescence signals fluctuated for the first 12 h of recording, and then, for most luciferin concentrations, light emission reached a stable plateau after 1 to 2 days with maximum bioluminescence being observed at 1.25 and 2.5 mM luciferin. At concentrations greater than 2.5 mM, light emission began to decline after 1 to 2 days, and after 3 days in these conditions, cells had a shriveled morphology indicating that luciferin may be cytotoxic at high levels in extracellular media.

We observed that ^app^K_m_ for luciferin changed significantly over the course of these recordings, beginning at just below 1 mM and falling to reach a stable level of ~30 µM, once bioluminescence levels stabilized, within less than 1 day (Fig. 2C, D). The ^app^K_m_ was sensitive to external temperature, as we observed it to increase by ~3-fold at 30 °C compared with 37 °C (Fig. 2E). The difference between the temperature-compensated K_m_ measured for Fluc in solution and the temperature dependence of ^app^K_m_ that we observed in cells is potentially significant since it may contribute to transient changes in circadian bioluminescent reporter activity during applied temperature cycles. To test this, we subjected U2OS cells stably expressing Fluc either constitutively, or under antiphasic promoters (Per2 and Bmal1), to 12-h:12-h temperature cycles between 30 and 37 °C. We observed acute changes in bioluminescence in every case that occurred immediately after the temperature transition but, unsurprisingly, such changes were not observed at constant temperature (Fig. 2F, Suppl. Fig S2B).

**Figure 3.**
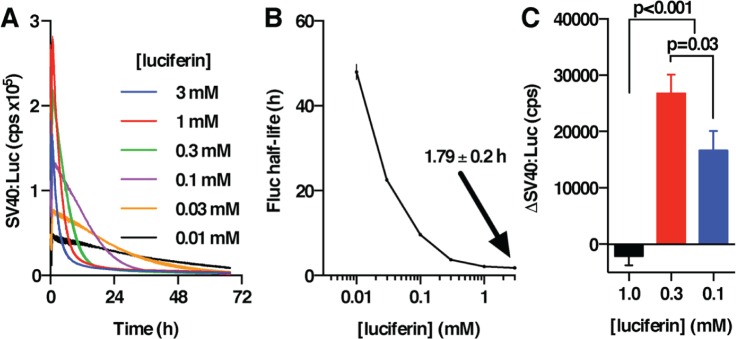
Stability of Fluc expressed constitutively in human U2OS cells. (A) The stability of Fluc catalytic activity (+50 µM CHX) is dependent upon extracellular luciferin concentration (mean ± SEM, *n* = 3); solid line shows 1-phase exponential decay fit (*R*^2^ ≥ 0.90). (B) Fluc half-life values from (A), for which *p* < 0.0001 by 1-way ANOVA. (C) Cells were preincubated with 1 mM luciferin for 40 h and then changed to fresh media with the indicated luciferin concentrations. After 1 h, NP40 detergent (or vehicle) was injected to a final concentration of 0.2% (mean ± SEM, *n* = 6); the change in light emission 30 min after injection (NP40 vs. respective vehicle control) is reported for 3 different concentrations of extracellular luciferin. Holm-Sidak tests *p* values are reported.

We next determined the half-life of cellular Fluc activity from the decay in bioluminescence of cells treated with the protein synthesis inhibitor cycloheximide (CHX, 50 µM). The curves were readily fit with a 1-phase exponential decay model (Fig. 3A), which yielded half-life values that increased from ~2 h at 1 mM luciferin in the media to ~2 days at 10 µM (Fig. 3B). At 37 °C, therefore, Fluc in cells is more stable, both thermally and catalytically, than was observed in solution (thermal half-life ~1.6 h, Fig. 1G; catalytic half-life ~0.2 h, Fig. 1D). Importantly, though, the inverse relationship between Fluc catalytic stability and luciferin concentration we observed in solution (Fig. 1E, F) was recapitulated in cellular assays (Fig. 3B), albeit with a greater effect size. This suggests that, particularly in the absence of destabilization motifs (e.g., PEST sequences), the primary determinant of Fluc enzymatic stability in cells is its catalytic turnover.

Several of our observations suggested to us that intracellular luciferin may not be in equilibrium with extracellular luciferin. In particular, ^app^K_m_ in cells (≥30 µM) is significantly greater than the K_m_ measured in solution (≤15 µM); ^app^K_m_ changes by an order of magnitude during the first day of incubation with cells; the temperature-dependence of ^app^K_m_ in cells is much greater than the equivalent change of K_m_ in solution; and the luciferin concentration–dependent variation in Fluc stability in solution is much less than that observed in cells. Luciferin is negatively charged at neutral pH and therefore requires active transport across the plasma membrane in order to become a substrate for intracellular Fluc (Patrick et al., 2014). Luciferin transport occurs primarily through members of the OATP family of organic anion antiporters in an electroneutral exchange for intracellular anions such as bicarbonate and glutathione (Roth et al., 2012; Patrick et al., 2014). OATP activity is essentially ubiquitous, although the expression and distribution of specific isoforms and family members vary between tissues and cell types, being highest in the brain, liver, lung, kidney, and testes (Roth et al., 2012; Patrick et al., 2014). We speculated, therefore, that in cell types where OATP expression is not highly abundant, the rate of luciferin transport might not always be sufficient to ensure that cytosolic luciferin is in saturating excess for Fluc.

To test this speculation, we preincubated cells for 40 h with 1 mM luciferin (to prevent the accumulation of active Fluc) and then changed them to fresh media containing 0.1, 0.3, or 1 mM luciferin. We then measured the change in bioluminescence upon addition of the detergent NP40, allowing rapid equilibration of luciferin over (former) compartments. We hypothesized that if intracellular luciferin were not in excess with 0.1 or 0.3 mM extracellularly, then we would observe an acute increase in Fluc activity upon detergent addition. Our observations agreed with the prediction (Fig. 3C), and, moreover, Fluc activity did not increase at 1 mM luciferin extracellularly, indicating that Fluc was already in the presence of saturating substrate prior to the introduction of NP40 at this higher concentration.

### Extracellular Luciferin Concentration Can Affect the Apparent Phase, Amplitude, and Period of Clock Gene Expression in Mammalian Cells

**Figure 4.**
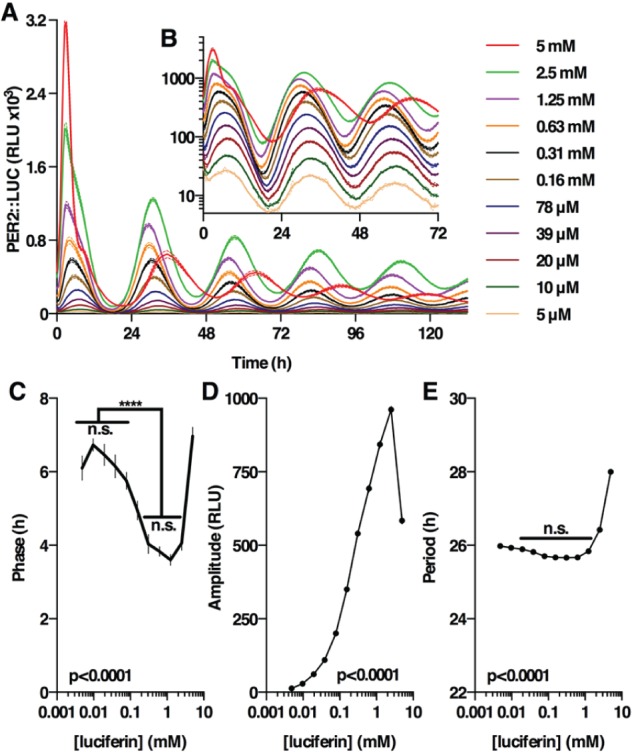
Effect of luciferin concentration on PER2::LUC reporter activity in mouse fibroblasts. (A) Bioluminescence recorded from PER2::LUC immortalized adult mouse fibroblasts incubated with a range of luciferin concentrations (mean ± SEM, *n* = 8). (B) Expansion of (A) and plotted on a log_10_ scale to highlight differences in apparent phase and amplitude. Significant differences were observed in apparent phase (C), amplitude (D), and circadian period (E) due to luciferin concentration. One-way ANOVA *p* values are reported as well as key summaries of Sidak’s multiple comparisons test.

To perform as a faithful reporter of gene expression, Fluc activity should not be limited by substrate availability. Our assays in solution and with cell-expressed Fluc indicated a clear relationship between Fluc catalytic turnover and enzymatic half-life. In consequence, if intracellular luciferin concentration is not in excess during a circadian bioluminescence assay, then the observed rhythm in light emission will be a function of not only Fluc but also luciferin concentration. A clear prediction, therefore, is that the lower the intracellular luciferin concentration, the later the apparent phase and the lower the amplitude the oscillation will manifest. To test this, we incubated immortalized fibroblasts isolated from adult PER2::LUC mouse lung tissue with a range of luciferin concentrations (Fig. 4A, B). We observed that the concentration of luciferin in the extracellular media had a highly significant effect on the phase and amplitude of PER2::LUC rhythms (Fig. 4C, D), with the earliest phases occurring at ~1 mM luciferin and becoming progressively later with decreasing luciferin concentration. At concentrations of luciferin ≥2.5 mM, we observed an unambiguous increase in circadian period (Fig. 4E), indicating that very high luciferin concentrations impinge upon the cellular timekeeping mechanism itself, very likely in a nonspecific fashion (see discussion).

The luciferin concentration–dependence of PER2::LUC amplitude and phase is consistent with our prediction based on observations that Fluc catalytic stability is increased at low luciferin concentration, in solution and in cells. Even though the differences in PER2::LUC phase and amplitude amounted to more than 2 h and ~100-fold, respectively, based on our prior findings (Figs. 1 [Fig fig2-0748730416668898]-3), there is no reason to believe that these arise from any effect on the abundance of the PER2::LUC fusion protein or the cellular clock mechanism itself, merely the fidelity with which Fluc activity reports molecular events associated with cellular timekeeping. To test this, we used a simple mathematical model to determine whether substrate-dependent differences in luciferase activity and catalytic stability are sufficient to account for our observations. Using half-life values of 2 h and 10 h that we observed at 0.1 mM and 1 mM luciferin, respectively (Fig. 3A, B), our model reproduced the ~2 h delay in the apparent phase of PER2::LUC that we observed experimentally, without any change in PER2::LUC protein abundance (Suppl. Fig S3).

### Extracellular Luciferin Concentration Can Affect the Period and Apparent Amplitude of Circadian Gene Expression in Organotypic SCN Slices

**Figure 5.**
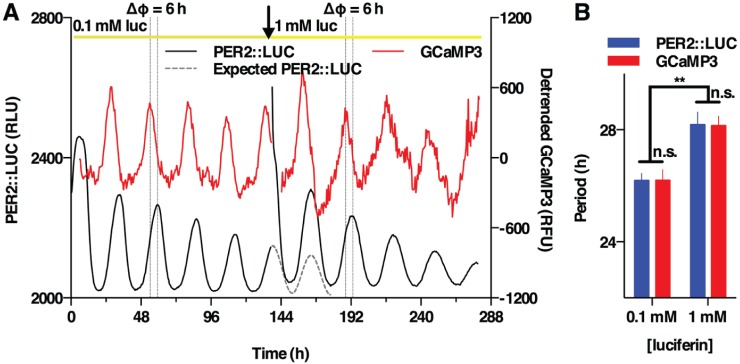
Effect of luciferin concentration on PER2::LUC reporter activity in organotypic SCN slice. (A) Representative recording from PER2::LUC SCN slices, lentivirally transduced with AAVs to express GCaMP3 calcium reporter (*n* = 3). No significant difference in phase was observed between the 2 reporters at 2 luciferin concentrations or between the actual and expected timing of the first PER2::LUC peak at 1 mM luciferin (*p* > 0.7, paired *t* test) or the amplitude of PER2::LUC (*p* = 0.14, paired *t* test). (B) A significant difference in period was observed between 0.1 mM and 1 mM luciferin (2-way ANOVA *p* = 0.0016, luciferin effect).

If the effect of extracellular luciferin concentration on molecular rhythms reported by Fluc was recapitulated in organotypic SCN slices, it would require careful reevaluation of many ex vivo experimental observations of the mammalian master clock that have used Fluc as the sole circadian reporter. However, PER2::LUC SCN slices, also cotransduced with GCaMP3 to detect intracellular calcium concentration in SCN neurons, showed no significant phase shifts in the transition from 0.1 mM to 1 mM luciferin and no changes in the phase angle between the PER2 and [Ca^2+^]_i_ peaks (Fig. 5A). We did observe, however, that the 10-fold increase in extracellular luciferin led to a significant increase in circadian period of both the PER2 and GCaMP3 reporters (Fig. 5B), similar to the effect of high extracellular luciferin on PER2::LUC rhythms in mouse fibroblasts.

## Discussion

Our experiments revealed that the half-life of luciferase in solution and in cells is predominantly determined by substrate turnover, as well as by thermal instability, and that physiologically relevant changes in the intracellular availability of PP_i_, CoASH, and Mg·ATP are unlikely to significantly affect the activity of Fluc expressed in the cytosol. Compared with Fluc in solution, Fluc in mammalian cells is protected from thermal denaturation by cellular chaperones (Nguyen and Bensaude, 1994), and slower rates of catalytic inactivation are also observed (Fig. 3A, B). Thus, a decrease in bioluminescence from cells expressing Fluc would only be likely to occur as a result of catalytic inactivation, a decrease in luciferin availability, reduced Fluc translation, or increased degradation. Only the latter two are of interest when Fluc is used as a reporter of circadian gene expression, whereas the first two are related to each other.

We also found that the initial activity of Fluc, with respect to its K_m_, is temperature compensated. This is unsurprising since, presumably, fireflies need to flash as brightly on cold evenings as warm ones. Coupled with other recent observations, however (Nakajima et al., 2005; Isojima et al., 2009; O’Neill et al., 2011), our findings do suggest that perhaps molecular temperature compensation is not particularly difficult to select for, given suitable evolutionary pressure. In contrast, we found that Fluc activity in mammalian cells was temperature-dependent and, moreover, that acute changes in temperature induced rapid changes in bioluminescence regardless of the promoter from which Fluc was expressed. A parsimonious explanation for our findings is that the kinetics of luciferin import are more temperature sensitive than those of its export, resulting in reduced steady-state intracellular luciferin concentration upon a temperature decrease that manifests as an increased ^app^K_m_.

Of particular relevance to circadian researchers, our observations suggest that if intracellular luciferin concentration is not in saturating excess, any genetic, pharmacological, or environmental perturbation that affects the cellular import of luciferin may lead to changes in bioluminescence that are unrelated to changes in circadian gene expression. Under these circumstances, in the context of clock-regulated luciferase reporters, changes in intracellular luciferin concentration would present themselves as an apparent change in circadian phase and amplitude of expression without any underlying biological clock correlate. At most, this could produce apparent phase shifts of several hours and amplitude differences of several fold. We observed this effect in cultured fibroblasts but not in SCN slices, suggesting differential efficiency of luciferin transport between the 2 models. We further note that if luciferin is not in saturating excess, any perturbation that elicits a gradual change in intracellular luciferin concentration over several days will manifest as an altered circadian period (albeit by ≤1 h) until intracellular luciferin concentration reaches a stable equilibrium. Our findings also clearly indicate that in longitudinal assays, when intracellular luciferin concentration is saturating, bioluminescence will correlate mostly closely with the amount of protein synthesized within the preceding 1 to 2 h, and not with total Fluc abundance.

We observed that at very high extracellular luciferin concentrations (>1 mM), luciferin does affect the period of the cellular clock itself, both in cultured fibroblasts as well as in SCN slices. Luciferin is negatively charged at neutral pH and so cannot enter the cell except through plasma membrane anion transporters. The best-characterized luciferin transporter is OATP1 (also known as SLC21A1/SLCO1A1), a member of the ubiquitously expressed family of solute carrier organic anion exchangers (Patrick et al., 2014). OATP1 imports luciferin in exchange for an intracellular dicarboxylate. Since intracellular dicarboxylates, such as succinate and glutathione, are critical to cellular metabolism, it is likely that the period lengthening observed at high luciferin concentrations is a nonspecific consequence resulting from the metabolic stress of intracellular dicarboxylate depletion.

Clearly, therefore, for longitudinal recordings from cultured cells and tissues, there exists an optimum concentration of extracellular luciferin under which circadian bioluminescence assays are performed, where intracellular luciferin concentration is sufficient to ensure that Fluc has the highest activity and shortest half-life. This will allow Fluc to report the actual phase and amplitude of changes in gene expression most closely. However, it is important not to use so much luciferin that it interferes (indirectly) with the timekeeping mechanism itself. Essentially, the optimum luciferin concentration is the highest one that does not elicit any increase in circadian period under constant conditions, and this should be determined empirically for each cell and tissue type. Importantly, we can infer that in cell types with low rates of luciferin import but with high expression levels of circadian transcriptional luciferase reporters, one would expect not to observe any rhythm in bioluminescence. Conversely, in tissues like the liver and kidney that express organic anion transporters such as OATP1 at very high levels, one would expect to detect much higher amplitude rhythms in circadian bioluminescence than are observed elsewhere, as has been observed in the liver and kidney in vivo (Tahara et al., 2012; Saini et al., 2013). Moreover, some OATP family members exhibit rhythmic transcript levels in mouse tissues (Zhang et al., 2014); whether this elicits any daily variation in luciferin import kinetics in vivo should also be considered.

Based on our experience, ~100 to 300 µM luciferin appears optimal for organotypic PER2::LUC SCN slices, ~300 to 500 µM is preferable with the human U2OS cell line expressing transcriptional luciferase fusions, and 0.5 to 1 mM performs best with PER2::LUC immortalized fibroblasts. Finally, we suggest that Fluc-expressing cells should be preincubated with luciferin for at least 24 h prior to longitudinal bioluminescence measurements to allow its intracellular concentration to reach steady-state equilibrium and prevent accumulation of active Fluc. We trust that our findings will be of use to readers in the design of their own Fluc-based circadian assays.

## Supplementary Material

Supplementary material
