# Supplementary material for: In-depth Characterization of Firefly Luciferase as a Reporter of Circadian Gene Expression in Mammalian Cells
Source: J Biol Rhythms. 2016 Oct 10;31(6):540–50. doi: 10.1177/0748730416668898 (PMC5117186; doi:10.1177/0748730416668898)
Supplement: Supplementary material [file Supplementary_Material.pdf]

Supplementary Figure 1

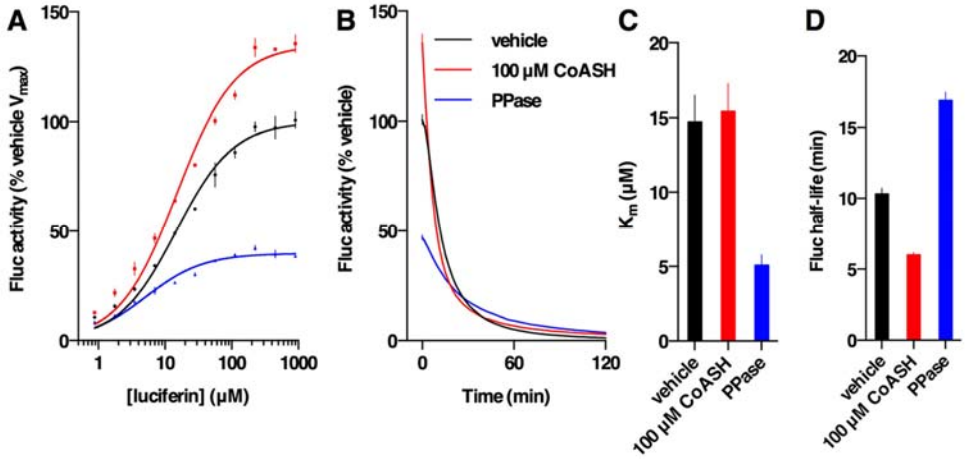

Figure S1. Modest effects of PP_i and CoASH on Fluc activity in solution at 37°C.

We repeated the determination of Fluc kinetics at 37°C, with respect to luciferin, in the presence or absence of inorganic pyrophosphatase (PPase) or 100 μM CoASH. (**A**) Both PPi and CoASH increase Fluc activity in terms of V_{max} (mean±SE, n=2). (**B**) Representative Fluc activity at 225 μM luciferin ± 100 μM CoASH or inorganic pyrophosphatase (PPase) (mean±SE, n=2). (**C**) K_m is affected by PP_i (p=0.02), but not CoASH (p=0.69), calculated from (A) and using Holm-Sidak's test. (**D**) At 225 μM luciferin, enzymatic half-life was significantly affected by PP_i, and 100 μM CoASH (mean±SE, n=2, p=0.001 and 0.002 vs. vehicle, respectively, by Holm-Sidak's test). These results further support that an inverse relationship exists between enzymatic turnover and catalytic half-life.

Supplementary Figure 2

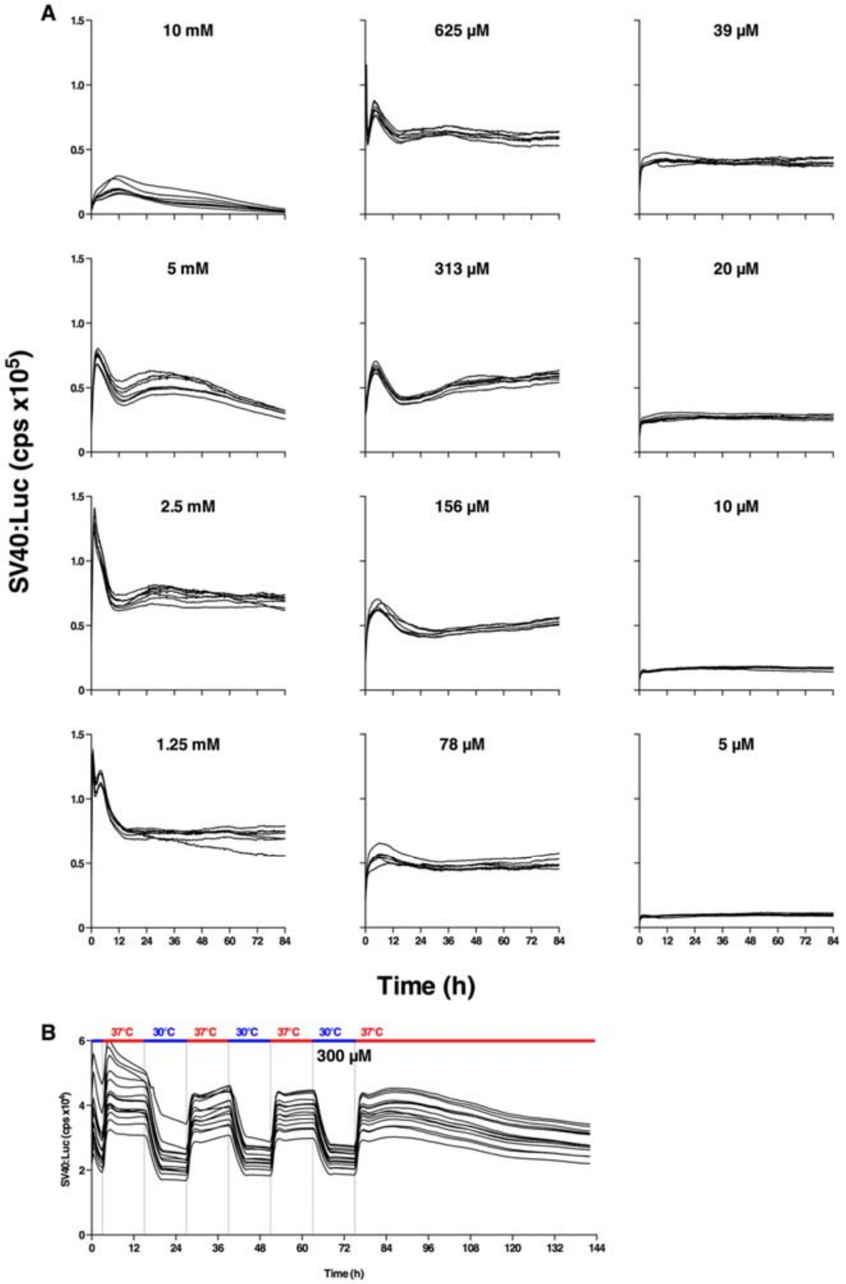

Figure S2. Bioluminescence from cells expressing Fluc constitutively varies with extracellular luciferin concentration and temperature.

(A) Raw bioluminescence traces are shown (n=7 per condition) from U2OS cells stably expressing Fluc from the SV40 promoter over a range of luciferin

concentrations, Mean±SEM for each condition is also presented in Figure 2B. (B) Raw bioluminescence traces are shown (n=15) from U2OS cells stably expressing Fluc from the SV40 promoter under an externally imposed 12 h:12 h 30°C:37°C temperature cycle. Note that this recording was made using an ALLIGATOR system, in which the CCD detector is located externally to the incubator system and cooled to constant -90°C, i.e., it is not subject to the temperature cycle imposed upon the cells. Also note that the incubator features no active cooling and therefore 30°C to 37°C transitions occur more rapidly than the converse.

Supplementary Figure 3

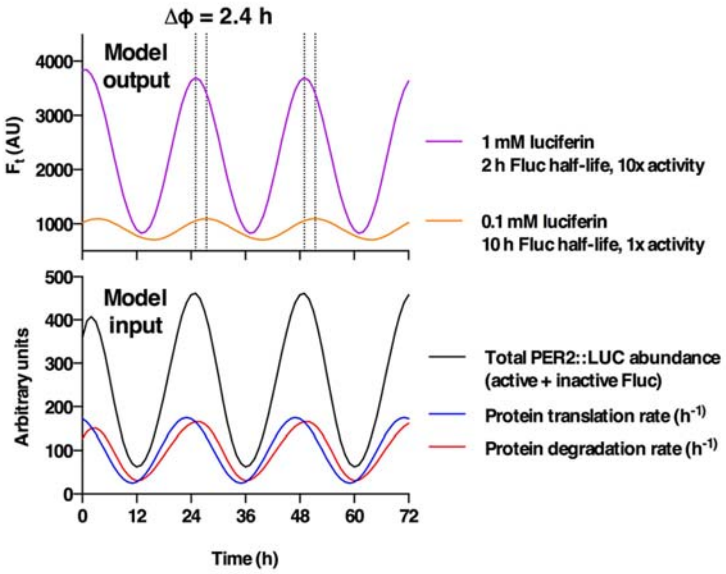

Figure S3. A simple model recapitulates [luciferin]-dependent differences in circadian phase.

The model employs no fitting, and simply assumes that PER2::LUC translation at time (t) is a function of *Per2*::*Luc* mRNA abundance, that PER2::LUC degradation rate follows one-phase exponential decay where the decay constant is defined by a sine wave with 24 h period, and that Fluc activity obeys one-phase exponential decay that varies with substrate concentration in the manner observed experimentally:

$P_t = P_{t-1} + S_t - D_t$

$B_t = S_t – D_t + B_{t-1}e^{-k}$

$S_t = 500*R_t$

$D_t = P_t * (1- e^{-Kt})$

$K_t = ln(2)/((A(sin((2π*t/24)+φ)))+H)$

$F_t = L(B_t)$

P_t is PER2::LUC protein abundance at t (175 at t=0)

S_t is total P translated in 1 h prior to t (in hours)

D_t is total P degradation in 1 h prior to t

B_t is active Fluc at t (200 for 1 mM, 800 for 0.1 mM at t=0)

R_t is *Per2*::*Luc* mRNA abundance at t (cosine wave with 24 h period)

K_t is the exponential decay constant at t

A is amplitude of the rhythm in PER2::LUC half-life (0.3)

φ is initial phase (1.6 h)

H is mean PER2::LUC half-life (1.3 h)

k is a luciferin-dependent Fluc activity decay constant (0.32 at 1 mM, 0.07 at 0.1 mM)

F_t is Fluc bioluminescence at t

L is a luciferin-dependent Fluc activity constant (activity at 1 mM is 10x 0.1 mM)
